# The prognostic significance of uric acid to albumin ratio in patients with aneurysmal subarachnoid hemorrhage following surgical clipping or endovascular interventions: insights from a large cohort study

**DOI:** 10.3389/fneur.2025.1648805

**Published:** 2026-01-12

**Authors:** Xielin Tang, Xiaoyi Wang, Bingcheng Zhu, Liangxue Zhou, Xiaolin Chen

**Affiliations:** 1 Department of Neurosurgery, The Affiliated Santai Hospital of North Sichuan Medical College, Mianyang, Sichuan, China; 2 Department of Neurosurgery, West China Hospital, Sichuan University, Chengdu, Sichuan, China; 3 Department of Neurosurgery, Mianyang Central Hospital, School of Medicine, University of Electronic Science and Technology of China (UESTC), Mianyang, Sichuan, China; 4 Department of Neurosurgery, Beijing Tiantan Hospital, Capital Medical University, Beijing, China

**Keywords:** uric acid, albumin, aneurysmal subarachnoid hemorrhage, prognosis, oxidative stress, inflammation

## Abstract

**Background:**

An increasing number of studies find uric acid to albumin ratio (UAR) plays an important role in predicting prognosis of cardiovascular disease. Nevertheless, the clinical significance of UAR in aneurysmal subarachnoid hemorrhage (aSAH) is unclear. This study aims to investigate the correlation between UAR and prognosis of aSAH.

**Methods:**

In this research, we retrospectively reviewed data of aSAH patients based on the LongTEAM registry. The primary endpoint is the functional outcome at 90 days after discharge and the secondary endpoint is the postoperative complications. Modified Rankin scale (mRS) was used to evaluate the functional outcome (mRS 0–2 was defined as favorable outcome and mRS 3–6 was defined as unfavorable outcome). Multivariate logistic regression was applied to evaluate the association between UAR and prognosis of aSAH. Receiver operating characteristic (ROC) curve was used to explore the predictive ability of UAR. Finally, patients were categorized into four groups based on the quartiles of UAR, and the association between UAR and in-hospital complications was assessed using multivariate logistic regression analysis.

**Results:**

A total of 937 patients were included in this study. After adjusting potential covariates, multivariate logistic regression showed that UAR was an independent risk factor of poor prognosis (OR (95%CI): 1.317 (1.235–1.405), *p* < 0.001). Furthermore, compared with reference quartile, patients in Q4 (UAR > 5.348) had a higher risk of postoperative major adverse cardiovascular events (OR (95%CI): 1.930 (1.286–2.898), *p* = 0.002), and postoperative pneumonia (OR (95%CI): 1.873 (1.210–2.898), *p* = 0.005). The ROC curve showed UAR had a satisfactory predictive performance (area under curve = 0.704).

**Conclusion:**

Elevated UAR levels were associated with high risk of unfavorable clinical outcomes, postoperative major adverse cardiovascular events, and pneumonia. UAR might be a simple, reliable, and cost-effective predictive marker for prognosis of aSAH.

**Clinical trial registration:**

https://clinicaltrials.gov/study/NCT04785976, NCT04785976.

## Introduction

Aneurysmal subarachnoid hemorrhage (aSAH) is a life-threatening neurosurgical disease with a sudden onset. The mortality rate of aSAH is nearly 50% and many survivors failed to recover independently (1). Hence, it is of great significance to find predictive markers for aSAH prognosis.

Serum uric acid (UA) is an end-product of purlin metabolic. According to previous research, serum UA was significantly associated with oxidative stress, inflammatory response, and endothelia dysfunction, which exacerbate the development of atherosclerosis and other cardiovascular disease (2). Furthermore, Ye et al. reported that high UA levels might increase the risk of aSAH (3). Serum albumin is also an important endogenous protein which can diminish oxidative stress and inflammatory response (4). The uric acid to albumin ratio (UAR) is a novel marker which has been identified to possess a superior predictive ability for evaluating the severity of oxidative stress, inflammation, and metabolic disorders than using UA or ALB alone (2). As so far, UAR has been demonstrated as an independent risk factor for poor prognosis and mortality in coronary artery disease, heart failure, carotid atherosclerosis, and other heart disease (2, 4, 5). A recent study also reported high UAR levels might increase the risk of lymph node metastasis in lung cancer (6). However, the association between UAR and prognosis of cerebrovascular disease remains unclear.

Given that oxidative stress and inflammation are critical pathological processes following aneurysm rupture which can significantly influence the clinical outcome of patients (7), the utility of UAR in predicting prognosis of aSAH is noteworthy. To determine whether UAR correlates with clinical outcomes of aSAH, we conducted this retrospective study.

## Methods

### Patients and study design

The information of patients with aSAH who presented to Beijing Tiantan Hospital from January 2015 to September 2022 was retrospectively reviewed. All the data were collected from the Long-term Prognosis of Emergency Aneurysmal Subarachnoid Hemorrhage (LongTEAM, ClinicalTrials.gov Identifier: NCT04785976) registry. We also collected informed consent from all included patients or their authorized representatives for approving clinical analysis. All participants underwent brain computed tomography (CT) scan to confirm the diagnosis of subarachnoid hemorrhage and CT angiography (CTA) or digital subtraction angiography (DSA) were applied to confirm the diagnosis of aneurysm. The inclusion criteria for this study included the following: (1) patients aged over 18 years; (2) presence of single aneurysm; (3) admission to hospital from the emergency department; (4) only receiving surgical clipping or endovascular treatment. The exclusion criteria included: (1) time interval exceeding 72 h from aneurysm rupture to surgery; (2) a prior history of aneurysm rupture; (3) a history of other neurosurgical conditions (such as brain tumor, arteriovenous malformation, cavernous hemangioma, Parkinson’s disease, hydrocephalus); (4) a history of intracranial endovascular treatment or craniotomy; (5) with functional or neurological disability resulting from other diseases; (6) liver, kidney, or other organ failure; (7) regularly taking UA lowering drugs in 6 months before admission; (8) failing to complete follow-up at 90 days after discharge.

### Data collection

The demographic data included age, sex, body mass index (BMI), time interval from rupture to admission, and length of stay. The preoperative clinical status included Graeb score, Subarachnoid Hemorrhage Early Brain Edema Score (SEBES) score, Hunt-Hess grade, World Federation of Neurosurgical Societies (WFNS) grade, modified Fisher Scale (mFS) grade, Glasgow coma scale (GCS), preoperative intraventricular hemorrhage (IVH), preoperative hydrocephalus, loss of consciousness, and seizure. The previous history included current smoking, current drinking, diabetes, hypertension, hyperlipemia, history of heart disease, history of antiplatelet, and history of anticoagulant. The in-hospital complications included rebleeding, intracranial infection, stress ulcer, urinary tract infection (UTI), major adverse cardiovascular events (MACE), delayed cerebral ischemia (DCI), deep vein thrombosis (DVT), anemia, and pneumonia. The fasting blood sample was collected in the first 24 h after treatment. Prior to blood sample collection, all patients were required to fast for at least 8 h. The postoperative laboratory test included, sodium (Na, mmol/L), potassium (K, mmol/L), Cl (mmol/L), aspartate aminotransferase (AST, U/L), alanine aminotransferase (ALT, U/L), total protein (Tp, g/L), globulin (Glb, g/L), triglyceride (Tg, mmol/L), cholesterol (Cho, mmol/L), gamma-glutamyl transferase (GGT, U/L), cholinesterase (CHE, IU/L), high-density lipoprotein (HDL, mmol/L), low-density lipoprotein (LDL, mmol/L), and homocysteine (Hcy, μmol/L). Moreover, the preoperative blood routine test, including white blood cell (WBC, 10^9^/L), lymphocyte (Ly, 10^9^/L), monocyte (Mono, 10^9^/L), neutrophil (Neu, 10^9^/L) were also collected. UAR was calculated as the following formula: UA (μmol/L)/ALB (g/L). BMI was calculated as the following formula: weight (kg)/ square of height (m^2^).

### Outcome assessment

The primary endpoint is the functional outcome assessed 90 days after discharge. The secondary endpoint is the incidence of in-hospital complications. At 90 days after discharge, patients were followed up via telephone or outpatient appointments. The modified Rankin Scale (mRS) was utilized to evaluate the functional outcome of aSAH patients. The favorable outcome was defined as mRS ranging from 0 to 2, while unfavorable outcome was defined as mRS ranging from 3 to 6. The detailed diagnostic criteria for in-hospital complications were presented in Supplementary Table 1.

### Statistical analysis

The continuous variables exhibiting a non-normal distribution were represented using median (interquartile range, IQR) and continuous variables with a normal distribution were represented using mean ± SD. The categorical variables were summarized as frequency (percentage). To evaluate differences in continuous variables, the nonparametric Mann–Whitney U test or Kruskal–Wallis test was employed. For analyzing statistical differences in categorical data, chi-square tests, continuity correction tests, or Fisher’s exact tests were applied. In accordance with the events per variable (EPV) principle, a maximum of 18 variables were included in the multivariate regression model for this study. Based on univariate analysis results and previous literature, the following variables were ultimately incorporated into the multivariate regression analysis: age, Graeb score 5–12, GCS, treatment modality, WFNS score 4–5, Hunt Hess score 4–5, max diameter of aneurysm, length of hospitalization, mFS score, IVH, loss of consciousness, hypertension, DVT, DCI, postoperative anemia, postoperative pneumonia, and preoperative WBC Furthermore, patients were grouped according to UAR quartiles, and three multivariate regression models were constructed using these selected variables to investigate the association between UAR and postoperative complications. Model 1 included age and gender. Model 2 included age, Graeb score 5–12, max diameter of aneurysm, mFS Score 3–4, IVH, GCS, treatment modality, WFNS score4-5, Hunt Hess score 4–5, loss of consciousness, hypertension,. Model 3 incorporated age, Graeb score 5–12, GCS, treatment modality, WFNS score 4–5, Hunt Hess score 4–5, loss of consciousness, hypertension, and preoperative WBC. The restricted cubic spline (RCS) model was employed to explore the dose–response relationships between UAR and prognosis of aSAH patients. To enhance quality of model fitting, the knots of RCS were determined based on the lowest Akaike Information Criterion (AIC) value. Receiver operating characteristic (ROC) curve and Delong test were applied to examine the predictive ability of UAR. R version 4.4.0 Statistical Software was used for all statistical analyses.

## Results

### Baseline characteristics

**Figure 1:**
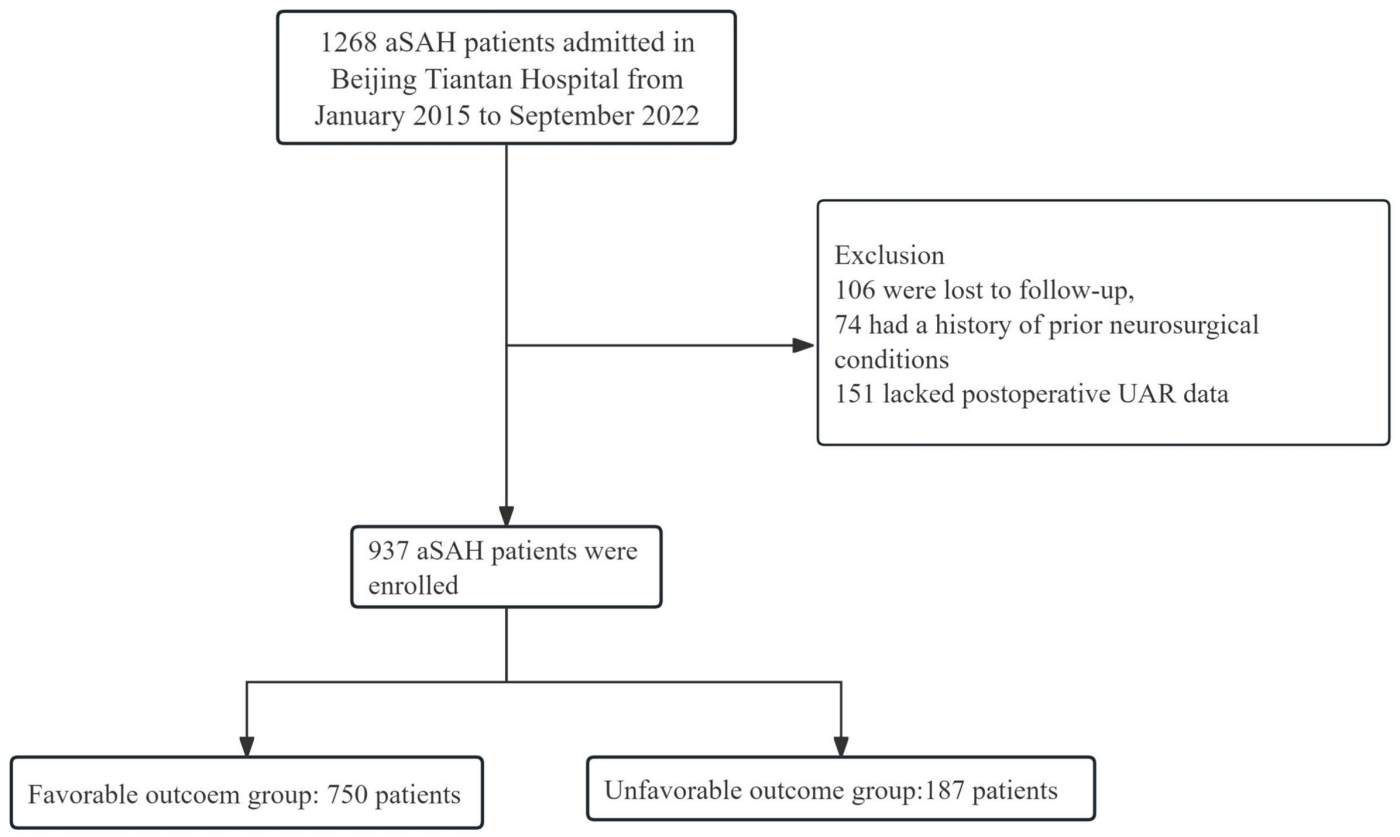
Flow diagram of the study participants.

A total of 1,268 aSAH patients were contained in LongTEAM registry. Among these patients, 106 patients were lost to follow-up, 74 patients had a history of prior neurosurgical conditions, and 151 patients lacked postoperative UAR data (Figure 1). Hence, three hundred and thirty-one ineligible patients were excluded and 937 patients with aSAH were included in this study. Among the 937 aSAH patients, 187 patients (19.96%) had unfavorable functional outcomes, 556 patients (59.34%) were female, 457 patients (48.77%) received endovascular treatment, and 480 patients (51.23%) received surgical clipping. All patients were categorized into four groups according to quartiles of UAR levels. The analysis of baseline characteristics was presented in Table 1. Compared with patients with favorable prognoses, those with unfavorable prognoses were older and had longer hospital stays. Regarding preoperative status, patients with poor prognoses exhibited lower preoperative GCS scores, and higher Graeb score, mFS score, WFNS score, and Hunt–Hess score. Additionally, a higher proportion of patients with unfavorable prognoses presented with preoperative intraventricular hemorrhage and loss of consciousness. In terms of medical history, hypertension and cardiac disease were more prevalent among patients with poor prognoses. With respect to postoperative complications, postoperative anemia, pneumonia, DCI, and DVT occurred more frequently in patients with unfavorable outcomes. Laboratory findings revealed that patients with poor prognoses had significantly elevated preoperative white blood cell count, preoperative monocyte count, preoperative neutrophil count, postoperative sodium, postoperative chloride, postoperative AST, and postoperative UAR, while postoperative LDL and TP were significantly decreased.

**Table 1:** The baseline characteristics of aSAH patients.

Variables	Favorable outcome (750)	Unfavorable outcome (187)	*p*
Demographic information			
Age, years	54.13 ± 11.06	58.65 ± 11.78	<0.001
BMI, kg/m^2^	24.93 ± 3.69	25.40 ± 4.07	0.128
Length of stay, days	12.00 (9.00, 16.00)	14.00 (10.00, 21.00)	<0.001
Rupture to admission, hours	24.00 (24.00, 48.00)	24.00 (24.00, 48.00)	0.700
Max diameter of aneurysm, diameters	5.20(4.00，8.00)	6.00 (4.37, 8.00)	0.030
Gender, *n* (%)			0.744
Female	447 (59.60)	109 (58.29)	
Male	303 (40.40)	78 (41.71)	
Preoperative clinical status			
GCS	13.68 ± 2.49	11.12 ± 4.03	<0.001
Graeb score 5–12, *n* (%)	48 (6.40)	30 (16.04)	<0.001
SEBES score 3–4, *n* (%)	358 (47.73)	95 (50.80)	0.452
mFS Score 3–4, *n* (%)	532 (70.93)	155 (82.89)	<0.001
IVH, *n* (%)	474 (63.20)	149 (79.68)	<0.001
Preoperative Hydrocephalus, *n* (%)	295 (39.33)	88 (47.06)	0.055
WFNS score 4–5, *n* (%)	123 (16.40)	91 (48.66)	<0.001
Hunt Hess score grade 4–5, *n* (%)	46 (6.13)	59 (31.55)	<0.001
Loss of consciousness, *n* (%)	210 (28.00)	92 (49.20)	<0.001
Seizure, *n* (%)	42 (5.60)	16 (8.56)	0.133
Treatment Modality, *n* (%)			0.897
Endovascular treatment	365 (48.67)	92 (49.20)	
Surgical clipping	385 (51.33)	95 (50.80)	
Previous history			
Current Smoking, *n* (%)	37 (15.74)	50 (21.37)	0.341
Current Drinking, *n* (%)	155 (20.67)	31 (16.58)	0.210
Diabetes, *n* (%)	68 (9.07)	22 (11.76)	0.263
Hypertension, *n* (%)	430 (57.33)	126 (67.38)	0.012
Hyperlipemia, *n* (%)	65 (8.67)	10 (5.35)	0.135
History of heart disease, *n* (%)	133 (17.73)	48 (25.67)	0.014
History of Antiplatelet, *n* (%)	4 (0.53)	2 (1.07)	0.757
History of anticoagulant, *n* (%)	29 (3.87)	9 (4.81)	0.557
In-hospital complications			
Postoperative rebleeding, *n* (%)	7 (0.93)	5 (2.67)	0.126
Postoperative intracranial infection, *n* (%)	90 (12.00)	31 (16.58)	0.095
Postoperative stress ulcer, *n* (%)	90 (12.00)	31 (16.58)	0.376
Postoperative UTI, *n* (%)	6 (2.55)	8 (3.42)	0.773
Postoperative Mace, *n* (%)	279 (37.20)	78 (41.71)	0.256
Anemia, *n* (%)	248 (33.07)	109 (58.29)	<0.001
Pneumonia, *n* (%)	232 (30.93)	127 (67.91)	<0.001
DCI, *n* (%)	174 (23.20)	96 (51.34)	<0.001
DVT, *n* (%)	252 (33.60)	98 (52.41)	<0.001
Laboratory test			
Preoperative WBC, 10^9^/L	12.78 ± 4.22	14.76 ± 4.83	<0.001
Preoperative Ly, 10^9^/L	0.96 (0.70, 1.33)	0.89 (0.67, 1.29)	0.701
Preoperative Mono, 10^9^/L	0.39 (0.26, 0.55)	0.47 (0.32, 0.67)	<0.001
Preoperative Neu, 10^9^/L	11.22 ± 4.05	13.09 ± 4.52	<0.001
Postoperative K, mmol/L	3.72 ± 0.43	3.66 ± 0.49	0.097
Postoperative Na, mmol/L	139.22 ± 4.56	142.06 ± 6.74	<0.001
Postoperative Cl, mmol/L	105.10 ± 5.57	108.47 ± 7.24	<0.001
Postoperative ALT, U/L	15.60 (11.60, 22.80)	17.00 (12.65, 25.80)	0.077
Postoperative AST, U/L	17.60 (14.00, 24.78)	22.00 (16.80, 32.85)	<0.001
Postoperative Tp, g/L	66.10 ± 6.68	64.01 ± 8.24	0.002
Postoperative GLB, g/L	26.71 ± 4.06	26.48 ± 4.76	0.534
Postoperative Tg, mmol/L	1.12 (0.82, 1.50)	1.18 (0.85, 1.54)	0.289
Postoperative Cho, mmol/L	4.58 ± 1.13	4.41 ± 1.02	0.060
Postoperative GGT, U/L	21.50 (14.80, 34.90)	22.90 (16.10, 37.45)	0.156
Postoperative CHE, IU/L	7924.97 ± 1944.56	7470.60 ± 2068.70	0.005
Postoperative HDL, mmol/L	1.36 ± 0.35	1.32 ± 0.35	0.163
Postoperative LDL, mmol/L	2.70 ± 0.94	2.52 ± 0.88	0.020
Postoperative Hcy, μmol/L	12.55 (9.96, 16.45)	12.10 (10.12, 16.80)	0.830
Postoperative UAR	3.89 (2.98, 4.94)	5.10 (3.88, 11.07)	<0.001

BMI, body mass index; SEBES, Subarachnoid Hemorrhage Early Brain Edema Score; WFNS, World Federation of Neurosurgical Societies; mFS, modified Fisher Scale; GCS, Glasgow coma scale; IVH, intraventricular hemorrhage; UTI, urinary tract infection; MACE, major adverse cardiovascular events; DCI, delayed cerebral ischemia; DVT, deep vein thrombosis; WBC, white blood cell; Ly, lymphocyte; Mono, monocyte; Neu, neutrophil; Na, sodium; K, potassium, Cl, Glu, glucose; AST, aspartate aminotransferase; ALT, alanine aminotransferase; Tp, total protein; Glb, globulin; Tg, triglyceride; Cho, cholesterol; GGT, gamma-glutamyl transferase; HDL, high-density lipoprotein; LDL, low-density lipoprotein; Hcy, homocysteine; mRS, modified Rankin scale.

### Association between UAR and functional outcome

The results of multivariate logistic regression were shown in Table 2.

**Table 2:** Univariate and multivariate logistic regression analysis of predictors for poor prognosis of aneurysmal subarachnoid hemorrhage patients.

Variables	Univariate analysis		Multivariate analysis	
	OR (95% CI)	*p*	OR (95% CI)	*p*
Age	1.038 (1.022–1.054)	<0.001	1.033 (1.014–1.054)	<0.001
Length of stay	1.074 (1.048–1.101)	<0.001	1.030 (0.998–1.062)	0.063
Max diameter of aneurysm	1.048 (1.004–1.094)	0.032	1.003 (0.950–1.058)	0.922
GCS	0.796 (0.759–0.835)	<0.001	1.066 (0.874–1.301)	0.526
Graeb score 5–12	2.795 (1.716–4.552)	<0.001	1.177 (0.613–2.260)	0.625
Treatment modality	0.979 (0.711–1.349)	0.897	1.049 (0.704–1.565)	0.813
mFS Score 3–4	1.985 (1.315–2.997)	0.001	0.632 (0.368–1.086)	0.097
IVH	2.283 (1.552–3.358)	<0.001	1.429 (0.862–2.368)	0.167
WFNS score 4–5	4.832 (3.419–6.828)	<0.001	2.936 (1.103–7.815)	0.031
Hunt Hess score grade 4–5	7.054 (4.593–10.834)	<0.001	2.041 (0.667–6.248)	0.211
Loss of consciousness	2.490 (1.794–3.457)	<0.001	0.764 (0.467–1.248)	0.283
Hypertension	1.537 (1.096–2.156)	0.013	1.224 (0.799–1.875)	0.353
History of heart disease	1.602 (1.098–2.338)	0.015		
Anemia	2.829 (2.038–3.927)	<0.001	1.659 (1.090–2.526)	0.018
Pneumonia	4.726 (3.351–6.666)	<0.001	2.154 (1.401–3.311)	<0.001
DCI	3.492 (2.503–4.873)	<0.001	2.771 (1.840–4.172)	<0.001
DVT	3.176 (1.573–3.010)	<0.001	0.989 (0.637–1.536)	0.960
Preoperative WBC	1.099 (1.061–1.138)	<0.001	1.074 (1.023–1.128)	0.004
Preoperative Mono	3.734 (2.073–6.725)	<0.001		
Preoperative Neu	1.105 (1.065–1.147)	<0.001		
Postoperative Na	1.106 (1.072–1.141)	<0.001		
Postoperative Cl	1.098 (1.067–1.129)	<0.001		
Postoperative AST	1.023 (1.012–1.033)	<0.001		
Postoperative Tp	0.959 (0.937–0.981)	<0.001		
Postoperative LDL	0.829 (0.937–0.981)	0.021		
UAR	1.331 (1.260–1.406)	<0.001	1.317 (1.235–1.405)	<0.001

**Figure 2:**
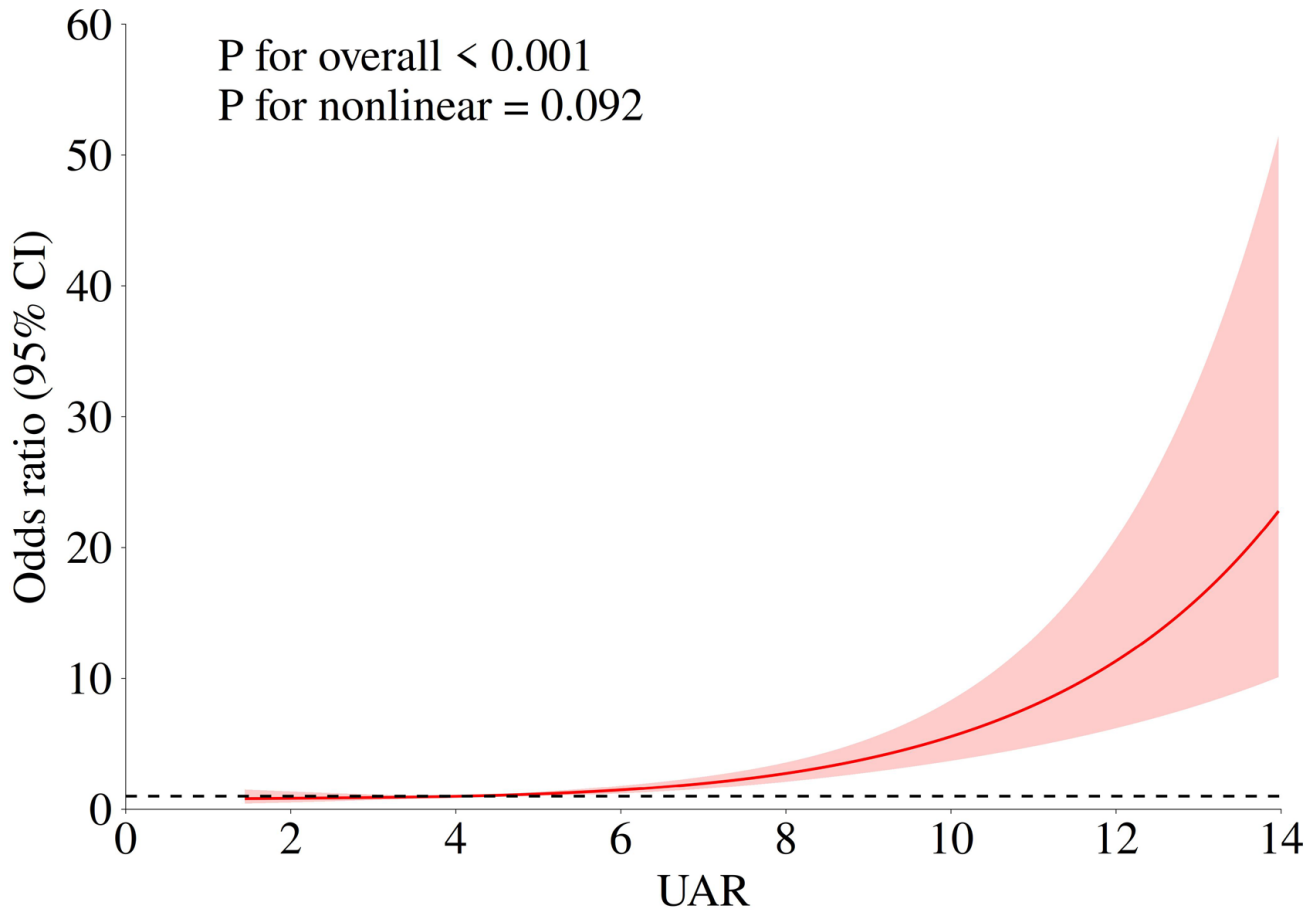
Association of UAR and functional outcome of aSAH patients. The adjusted model: age, max diameter of aneurysm, IVH, Graeb score 5–12, GCS, treatment modality, WFNS score4-5, Hunt Hess score 4–5, loss of consciousness, hypertension, postoperative DVT, postoperative anemia, postoperative pneumonia, DCI, and preoperative WBC.

In multivariate analysis, age (OR (95%CI): 1.033 (1.14–1.054), *p* < 0.001), WFNS 4–5 (OR (95%CI): 2.936 (1.103–7.815), *p* = 0.031), postoperative pneumonia (OR (95%CI): 2.771 (1.840–4.072), *p* < 0.001), postoperative DCI (OR (95%CI): 2.154 (1.401–3.311), *p* < 0.001), postoperative anemia (OR (95%CI): 1.659 (1.090–2.526), *p* = 0.018), preoperative WBC (OR (95%CI): 1.074 (1.023–1.128), *p* = 0.004)and UAR (OR (95%CI): 1.317 (1.235–1.405), *p* < 0.001) were identified as independent risk factors for poor outcomes in the multivariate logistic regression. The RCS model indicated that UAR had a linear relationship with an unfavorable functional outcome, suggesting elevated UAR levels may significantly increase the risk of poor prognosis (p for non-linear = 0.103, Figure 2).

### The receiver operating curve of UAR

**Figure 3:**
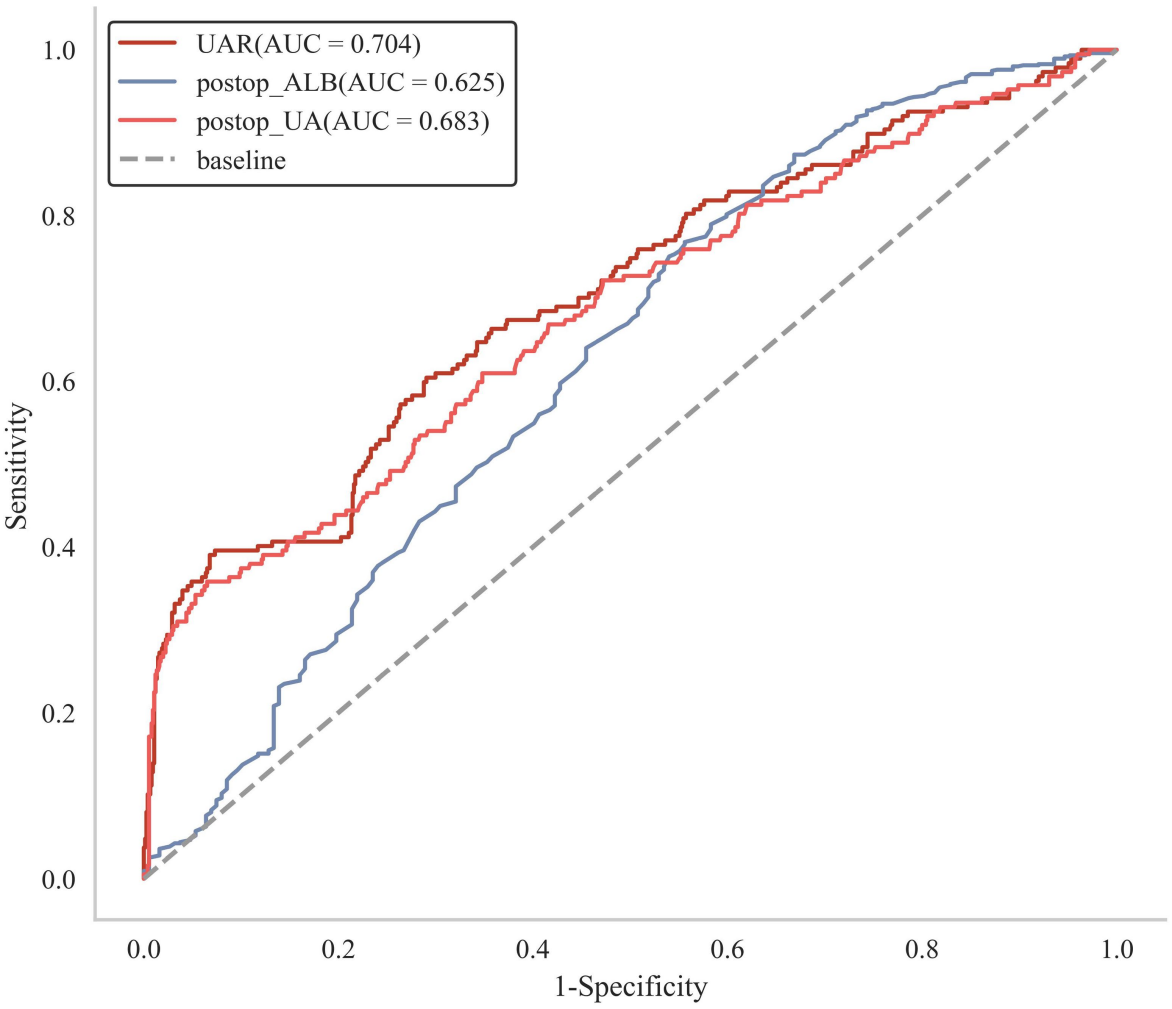
Receiver operating characteristic curve (ROC) of the UAR for the prediction of functional outcome.

The ROC curve of UAR for predicting functional outcome was depicted in Figure 3. The area under curve (AUC) of UAR was 0.704, indicating satisfactory performance for predicting the functional outcome of aSAH. The optimal cut-off value of UAR was 8.018, with a sensitivity of 0.396 and a specificity of 0.927 (Table 3). Furthermore, we evaluated the capabilities of UA (AUC = 0.683, sensitivity = 0.358, specificity = 0.934) and ALB (AUC = 0.625, sensitivity = 0.344, specificity = 0.768) for predicting unfavorable function outcomes. The Delong test demonstrated that UAR exhibited a superior prognostic predictive ability than UA (*p* = 0.0035) and ALB (*p* = 0.046, Supplementary Table 2).

**Table 3:** The prediction ability of UAR for the unfavorable outcome of aneurysm patients.

Characteristic	Area under the curve	Cut-off value	Sensitivity	Specificity	Youden
UAR	0.704	8.018	0.396	0.927	0.322
ALB	0.625	36.200	0.344	0.768	0.212
UA	0.683	301.200	0.358	0.934	0.293

UAR, uric acid to albumin ratio; ALB, albumin; UA, uric acid.

### Association between UAR and postoperative complications

**Figure 4:**
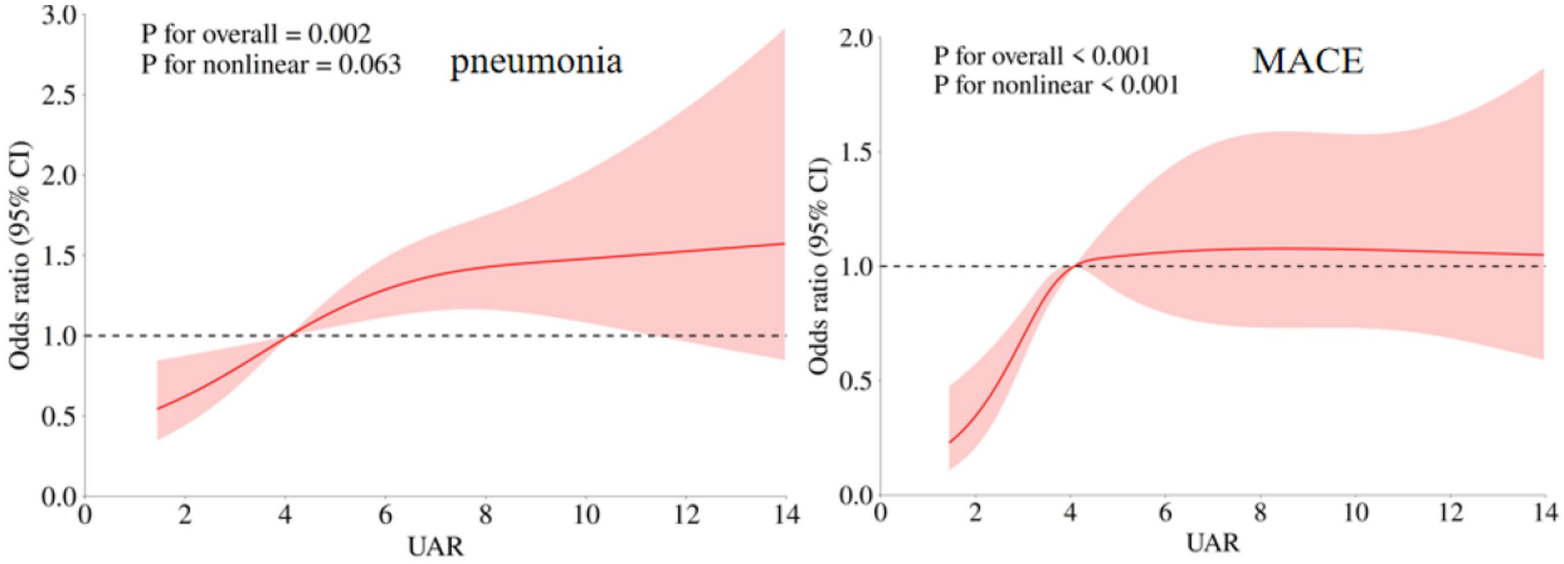
Association of UAR and in-hospital complications (postoperative MACE and pneumonia) of aSAH patients. The adjusted model: age, max diameter of aneurysm, IVH, Graeb score 5–12, GCS, treatment modality, WFNS score 4–5, Hunt Hess score 4–5, loss of consciousness, hypertension, and preoperative WBC.

In this study, pneumonia was the most common in-hospital complication with an incidence rate of 38.32% (359/937), and the second prevalent complication was MACE (357/937, 338.10%). The comparisons of UAR among patients with or without in-hospital complications were presented in Supplementary Table 3. Our findings indicated that postoperative UAR levels were significantly higher in patients who experienced MACE than those who did not (4.39 (3.40, 5.75) vs. 3.91 (2.92, 5.14), *p* < 0.001). Similarly, patients with pneumonia exhibited elevated UAR levels (4.45 (3.30, 6.37) vs. 3.92 (2.95, 4.96), *p* < 0.001). Furthermore, those who experienced DCI also showed higher postoperative UAR levels (4.26 (3.29, 5.50) vs. 3.96 (3.03, 5.25), *p* = 0.015). After adjusting for potential covariates, multivariate logistic regression demonstrated that patients in fourth quartile of a significant higher risk of MACE (OR (95%CI): 1.930 (1.286–2.898), *p* = 0.002) and pneumonia (OR (95%CI): 1.873 (1.210–2.898), *p* = 0.005, Table 4). The RCS analysis revealed that there was a significant non-linear association between UAR and MACE (p for overall <0.001, p for non-linear <0.001), while a linear relationship was observed between UAR and pneumonia (p for overall = 0.002, p for non-linear = 0.063, Figure 4). No significant association between other in-hospital complications and UAR (Table 5).

**Table 4:** The association between baseline UAR level and the risk of in-hospital complications.

In-hospital complications	Model 1		Model 2		Model 3	
	OR (95% CI)	*p*	OR (95% CI)	*p*	OR (95% CI)	*p*
Postoperative MACE						
Q1 (<3.063)	1.0(Ref)		1.0(Ref)		1.0(Ref)	
Q2 (3.063–4.079)	1.386 (0.933–2.061)	0.106	1.560 (1.041–2.339)	0.043	1.454 (0.969–2.180)	0.071
Q3 (4.070–5.348)	1.810 (1.219–2.686)	0.003	2.034 (1.363–3.035)	<0.001	1.847 (1.232–2.770)	0.003
Q4 (>5.348)	1.927 (1.297–2.864)	0.001	2.163 (1.447–3.233)	<0.001	1.930 (1.286–2.898)	0.002
Postoperative anemia						
Q1 (<3.063)	1.0(Ref)		1.0(Ref)		1.0(Ref)	
Q2 (3.063–4.079)	1.217 (0.829–1.785)	0.316	1.230 (0.828–1.827)	0.305	1.445 (0.962–2.169)	0.076
Q3 (4.070–5.348)	1.284 (0.869–1.896)	0.209	1.148 (0.769–1.714)	0.499	1.503 (0.989–2.286)	0.056
Q4 (>5.348)	1.517 (1.025–2.244)	0.037	1.051 (0.703–1.572)	0.807	1.412 (0.924–2.157)	0.110
Postoperative delayed cerebral ischemia						
Q1 (<3.063)	1.0(Ref)		1.0(Ref)		1.0(Ref)	
Q2 (3.063–4.079)	1.049 (0.690–1.594)	0.823	1.115 (0.728–1.705)	0.617	1.111 (0.724–1.706)	0.629
Q3 (4.070–5.348)	1.420 (0.941–2.141)	0.094	1.455 (0.954–2.186)	0.082	1.432 (0.939–2.186)	0.096
Q4 (>5.348)	1.340 (0.885–2.028)	0.167	1.216 (0.800–1.48)	0.361	1.218 (0.793–1.869)	0.367
Postoperative deep vein thrombosis						
Q1 (<3.063)	1.0(Ref)		1.0(Ref)		1.0(Ref)	
Q2 (3.063–4.079)	0.688(0.460–1.029)	0.068	0.731 (0.482–1.109)	0.141	0.771 (0.506–1.173)	0.225
Q3 (4.070–5.348)	1.072 (0.722–1.592)	0.731	1.077 (0.714–1.625)	0.723	1.178 (0.774–1.795)	0.445
Q4 (>5.348)	0.986 (0.661–1.471)	0.945	0.851 (0.562–1.291)	0.449	0.914 (0.597–1.401)	0.681
Postoperative intracranial infection						
Q1 (<3.063)	1.0(Ref)		1.0(Ref)		1.0(Ref)	
Q2 (3.063–4.079)	1.166 (0.661–2.058)	0.824	1.188 (0.663–2.129)	0.562	1.190 (0.664–2.143)	0.559
Q3 (4.070–5.348)	1.278 (0.730–2.240)	0.472	1.326 (0.748–2.344)	0.335	1.330 (0.742–2.360)	0.330
Q4 (>5.348)	0.919 (0.514–1.643)	0.776	0.820 (0.445–1.511)	0.525	0.821 (0.446–1.512)	0.527
Pneumonia						
Q1 (<3.063)	1.0(Ref)		1.0(Ref)		1.0(Ref)	
Q2 (3.063–4.079)	1.236 (0.834–1.832)	0.291	1.609 (1.047–2.473)	0.030	1.580 (1.027–2.432)	0.037
Q3 (4.070–5.348)	1.493 (1.008–2.211)	0.045	1.774 (1.152–2.733)	0.009	1.697 (1.098–2.621)	0.017
Q4 (>5.348)	2.012 (1.361–2.975)	<0.001	1.872 (1.211–2.893)	0.006	1.873 (1.210–2.898)	0.005
Postoperative rebleeding						
Q1 (<3.063)	1.0(Ref)		1.0(Ref)		1.0(Ref)	
Q2 (3.063–4.079)	0.333 (0.031–3.282)	0.349	0.382 (0.038–3.923)	0.418	0.389 (0.038–3.998)	0.427
Q3 (4.070–5.348)	0.340 (0.109–4.211)	0.677	0.745 (0.114–4.881)	0.759	0.747 (0.113–4.936)	0.762
Q4 (>5.348)	2.042 (0.482–8.655)	0.333	1.213 (0.243–6.604)	0.814	1.246 (0.246–6.321)	0.791
Postoperative stress ulcer						
Q1 (<3.063)	1.0(Ref)		1.0(Ref)		1.0(Ref)	
Q2 (3.063–4.079)	1.255 (0.813–1.936)	0.305	1.389 (0.889–2.169)	0.149	1.429 (0.912–2.238)	0.119
Q3 (4.070–5.348)	1.161 (0.747–1.806)	0.507	1.219 (0.773–1.922)	0.394	1.293 (0.817–2.047)	0.273
Q4 (>5.348)	1.153 (0.838–1.585)	0.131	1.269 (0.808–1.990)	0.301	1.281 (0.815–2.014)	0.283
Postoperative urinary tract infection						
Q1 (<3.063)	1.0(Ref)		1.0(Ref)		1.0(Ref)	
Q2 (3.063–4.079)	1.503 (0.510–4.428)	0.460	1.564 (0.525–4.659)	0.422	1.549 (0.520–4.620)	0.432
Q3 (4.070–5.348)	2.031 (0.715–5.769)	0.183	2.218 (0.769–6.390)	0.140	2.170 (0.749–6.289)	0.153
Q4 (>5.348)	1.788 (0.614–5.208)	0.287	1.579 (0.532–4.691)	0.411	1.582 (0.532–4.702)	0.409

Model 1: age, gender.

Model 2: age, Graeb score 5–12, max diameter of aneurysm, mFS Score 3–4, IVH, GCS, treatment modality, WFNS score 4–5, Hunt Hess score 4–5, loss of consciousness, and hypertension.

Model 3: age, max diameter of aneurysm, IVH, Graeb score 5–12, GCS, treatment modality, WFNS score4-5, Hunt Hess score 4–5, loss of consciousness, hypertension, and preoperative WBC.

**Table 5:** Associations of UAR with postoperative complications at 90 days follow-up.

Outcomes	Knots	P-overall	P-non-linear
Postoperative MACE	4	<0.001	<0.001
Anemia	3	0.142	0.106
Pneumonia	3	0.002	0.063
Delayed cerebral ischemia	5	0.103	0.159
Postoperative rebleeding	4	0.407	0.285
Postoperative intracranial infection	5	0.261	0.155
Postoperative stress ulcer	4	0.213	0.182
Postoperative UTI	3	0.706	0.420
Postoperative DVT	3	0.958	0.781

MACE, major adverse cardiac event; UTI, urinary tract infection; DVT, deep venous thrombosis; DCI, delayed cerebral ischemia.

## Discussion

This investigation reveals that UAR levels can predict the functional outcome of aSAH patients at 90 days after discharge and the incidence of in-hospital complications. A positive and linear association is found between postoperative UAR levels and unfavorable functional outcome. ROC analysis demonstrated UAR possessed a satisfactory prognostic predictive performance. In addition, high UAR levels were identified to increase the risk of postoperative MACE and pneumonia.

UAR was demonstrated to be a novel, reliable and cost-effective predictive factor for the mortality and prognosis of coronary artery disease, acute aortic dissection, pulmonary arterial and other cardiovascular diseases (8–10). Recently, a retrospective study reported that high UAR levels might increase the risk of lymph node metastasis in lung cancer (6). Nevertheless, the association between UAR and neurosurgery diseases is unclear. To the best of our knowledge, this is the first research identifying the high UAR level is a unfavorable prognostic factor for aSAH patients. The underlying mechanism linking UAR and poor prognosis was unclear. Potential explanations for this phenomenon may be the association between UAR and early brain injury (EBI) after aneurysm rupture. EBI is a critical process in the first 72 h following the onset of aSAH. This process encompasses oxidative stress, inflammation, cytotoxicity and other important pathological responses, which can significantly influence the clinical outcome of patients (11, 12). In 2021, Dodd et al. established an subarachnoid hemorrhage (SAH) animal model and found the nucleotide-binding oligomerization domain-like receptor protein 3 (NLRP3), an important protein complex positively regulating the inflammatory pathways, played a critical role in EBI following subarachnoid hemorrhage (13). In this animal model, NLRP3 was demonstrated to be able to promote the expression of interleukin-1β (IL-1β), increasing the inflammation level and severity of EBI. Meanwhile, Seifar and his colleagues found that UA could activate NLRP3 through NF-κB/IKK/p65 signal pathway (14). After aneurysm rupture, UA in blood might be released into subarachnoid space and cerebral fluid (CSF). Hence, we consider that excessive UA entering CSF will increase the expression of NLRP3, which induces a more sever inflammatory response and aggravates EBI. Besides the inflammation, the paradox role of UA in oxidative stress is also worth discussing. For one thing, UA is considered to have anti-oxidative stress for it can effectively scavenge radicals (15). In stroke experimental models, UA was also identified to be an antioxidant with neuroprotective effect (16). For another thing, UA is also identified to have a pro-oxidative stress function. In 2021, a cross-sectional study based on MedCity21 health examination registry reported a positive correlation between serum UA levels and the derivative of reactive oxygen metabolites (d-ROMs), a blood marker which can reflect oxidative stress reliably (17). This finding indicated that UA could promote oxidative stress through increasing the production of reactive oxygen species. In addition, a meta-analysis identified UA had no significant relationship with functional recovery at 3 months after onset for ischemic stroke patients (18). In 2021, Gong et al. (19) also reported that high UA levels increased the risk of neurological deterioration in patients with intracranial hemorrhage. Hence, the neuroprotective function of UA for cerebral vascular diseases required future validation. As for ALB, previous studies had confirmed that it played a significant role in inhibiting inflammatory response and oxidative stress (20, 21). Hence, as a combination of ALB and UA, high UAR may be associated with more severe inflammation and oxidative stress status, contributing to a poor prognosis.

Another noteworthy finding of our research is that elevated UAR levels might increase the risk of postoperative MACE and pneumonia. There were a few previous investigations that explored the risk factors associated with postoperative MACE. For instance, in 2023, Jia et al. (22) found insular cortex Hounsfield units could predict neurocardiogenic injury after aSAH. In 2024, Wang et al. demonstrated a correlation between lower intraoperative mean arterial pressure and higher incidence of cardiovascular events after aSAH treatment (23). The possible explanation for the association between UAR and postoperative MACE is that elevated UAR indicates more severe oxidative stress, which might result in endothelial dysfunction, increasing the risk of cardiovascular atherosclerosis and other heart diseases (5). As for postoperative pneumonia, two predictive models based on machine learning had been reported (24, 25). UAR may contribute to establishing a more reliable and accurate predictive model for postoperative pneumonia.

Given that the significant association between high UAR level and poor prognosis of aSAH, UA and ALB may be novel therapeutic targets for aneurysm patients. A clinical trial conducted in 2012 reported that ALB in dose of 1.25 g/kg/day was safe and facilitated neurology function recovery for aSAH patients (26). However, the optimal levels of UA for aSAH patients remain unclear (18). This study demonstrated that the cut-off value of UAR for predicting functional outcome of aSAH patients was 8.018. Further prospective randomized clinical trials are necessary to establish the optimal UAR level for the clinical management of aSAH.

There are some limitations in our study. First, this is a retrospective and single center study, which limited a deeper analysis for UAR. Prospective studies with multi-center study are necessary for confirming predictive role of UAR for aSAH prognosis among different ethnic groups or populations. Second, there remained some confounding were failed to be collected, such as alcohol level in blood, and socioeconomic status, which might induce potential bias to this study. Third, in this study, preoperative blood samples from aSAH patients were collected in the emergency ward. These samples were only subjected to basic blood routine tests, and UAR indicators could not be obtained. Hence,alteration of UAR during hospitalization was lost, limiting the analysis for cumulative and causal relationship between UAR and aSAH prognosis. Fourth, in this study, functional outcome was evaluated at 90 days after discharge. The association between UAR and long-term clinical outcome of aSAH still needs to be identified. Finally, the hypothesized explanations for mechanism between UAR and aSAH prognosis were oxidative stress and inflammation. Future fundamental experiments are necessary to validate this hypothesis.

## Conclusion

Elevated UAR levels were associated with high risk of unfavorable clinical outcome, postoperative major adverse cardiovascular events, and pneumonia. UAR might be a simple, reliable, and cost-effective predictive marker for prognosis of aSAH patients.

## Data Availability

The raw data supporting the conclusions of this article will be made available by the authors, without undue reservation.
