# Supplementary material for: The prognostic significance of uric acid to albumin ratio in patients with aneurysmal subarachnoid hemorrhage following surgical clipping or endovascular interventions: insights from a large cohort study
Source: Front Neurol. 2026 Jan 12;16:1648805. doi: 10.3389/fneur.2025.1648805 (PMC12832354; doi:10.3389/fneur.2025.1648805)
Supplement: Supplementary file 1 [file Table_1.DOC]

Supplemental Table 1. Definitions of in-hospital complications.

| **In-hospital complications** | **Definitions** |
| --- | --- |
| Rebleeding | Sudden clinical deterioration occurring during postoperative hospital stay with signs of increased bleeding on serial CT scans. |
| Delayed cerebral ischemia | New focal neurological deficit or global neurological deterioration (a drop of ≥ 2 points on the Glasgow Coma Scale [GCS]) lasting > 2 hours, after exclusion of intracranial hemorrhage, hydrocephalus, seizures, metabolic derangements, and infection, with or without radiological signs of cerebral vasospasm. |
| Intracranial infection | (1) The patient had clinical manifestations of various intracranial infections such as postoperative fever, headache, or neck stiffness. (2) The patient’s cerebrospinal fluid test showed inflammatory index changes, which met one of the following: white blood cell count > 0.01 × 10^9^ /L; cerebrospinal fluid protein > 4.50 g/L; cerebrospinal fluid glucose < 2.50 mmol/L. (3) The peripheral blood white blood cell count was > 10 × 10^9^ /L. |
| Major adverse cardiac event | The composite of total death; myocardial infarction; stroke, hospitalization because of heart failure; and revascularization, including percutaneous coronary intervention, and coronary artery bypass graft. |
| Stress ulcer bleeding | No previous history of peptic ulcer or peptic hemorrhage, and after treatment for aSAH, patients were tested positive by fecal occult blood test. |
| Urinary tract infection | Clinical symptoms of urinary tract infection or positive urine culture. |
| Anemia | In men, a hemoglobin of less than 130 to 140 g/L (13 to 14 g/dL); in women, it is less than 120 to 130 g/L (12 to 13 g/dL). |
| Pneumonia | Description of clinical indications or positive chest radiograph. |
| Deep vein thrombosis | Clinical diagnosis of deep vein thrombosis supported by ultrasound or venography. |

Supplemental Table 2 . Delong test for ALB, UA and UAR

| Comparison of AUC | |
| --- | --- |
| UAR vs ALB | |
| Difference between areas | 0.0789 |
| Z statistic | 2.833 |
| Significant level | P=0.0046 |
| UAR vs UA |  |
| Difference between areas | 0.0212 |
| Z statistic | 2.919 |
| Significant level | P=0.0035 |

Supplemental Table 3 The comparison of UAR between patients with or without in-hospital complications

| In-hospital complications | Yes | No | p |
| --- | --- | --- | --- |
| Rebleeding | 5.56 (3.40, 8.78) | 4.07 (3.06, 5.30) | 0.172 |
| Delayed cerebral ischemia | 3.96 (3.03, 5.25) | 4.29 (3.26, 5.50) | **0.015** |
| Intracranial infection | 4.20 (3.23, 5.29) | 4.04 (3.05, 5.36) | 0.359 |
| Major adverse cardiac event | 4.39 (3.40, 5.75) | 3.91 (2.92, 5.14) | **<.001** |
| Stress ulcer bleeding | 4.20 (3.20, 5.85) | 4.03 (3.03, 5.24) | 0.106 |
| Urinary tract infection | 4.07 (3.06, 5.34) | 4.15 (3.50, 5.75) | 0.443 |
| Anemia | 4.13 (3.12, 5.64) | 4.03 (3.06, 5.25) | 0.358 |
| Pneumonia | 4.45 (3.30, 6.37) | 3.92 (2.95, 4.96) | **<.001** |
| Deep vein thrombosis | 4.02 (3.11, 5.24) | 4.19 (3.03, 5.50) | 0.256 |
